# Acalabrutinib‐related second primary malignancies and nonmelanoma skin cancers in patients with chronic lymphocytic leukaemia (CLL): A systematic review and meta‐analysis of randomised controlled trials (RCTs)

**DOI:** 10.1002/jha2.146

**Published:** 2020-12-04

**Authors:** Thura W. Htut, Myat M. Han, Kyaw Z. Thein

**Affiliations:** ^1^ Department of Haematology Aberdeen Royal Infirmary Foresterhill Health Campus Aberdeen United Kingdom; ^2^ Division of Hematology and Medical Oncology Oregon Health and Science University/ Knight Cancer Institute Portland Oregon United States; ^3^ Department of Investigational Cancer Therapeutics The University of Texas MD Anderson Cancer Center Houston Texas United States

**Keywords:** acalabrutinib, chronic lymphocytic leukaemia, meta‐analysis, nonmelanoma skin cancers, second primary malignancies

## Abstract

Acalabrutinib is a second generation Bruton's tyrosine kinase inhibitor and was recently approved in the treatment of chronic lymphocytic leukaemia. We undertook a systematic review and meta‐analysis of randomised controlled trials to determine the risks of acalabrutinib‐related second primary malignancies (SPM) and nonmelanoma skin cancers (NMSC). The incidence of SPM was 4.7% higher in the acalabrutinib arm compared to control arm with risk ratio (RR) of 1.76 (5.32 vs 3.2 per 100 person‐years). Notably, NMSC was the most common SPM, and the incidence was 2.56 per 100 person‐years in the acalabrutinib group versus 1.12 per 100 person‐years in the control group (RR 2.43). Long‐term follow‐up and future studies are necessary to define the actual relationship and their risk factors.

## 1 INTRODUCTION

Acalabrutinib is a second generation and irreversible oral inhibitor of Bruton's tyrosine kinase (BTK) [3]. BTK is overly expressed on the surface of clonal B cells in chronic lymphocytic leukaemia (CLL) [12]. BTK inhibitors have revolutionized the treatment paradigm for patients with CLL over the recent years due to its survival benefits [2]. Acalabrutinib selectively inhibits BTK with significantly less off‐target inhibition of other kinases including Tec protein tyrosine kinase (TEC), endothelial growth factor receptor, and interleukin‐2‐inducible T cell kinase (ITK) [3]. T lymphocytes and natural killer (NK) cells are the cornerstones of cancer surveillance due to their effect on early recognition and cytotoxic killing of cancerous cells. CLL is associated with T and NK cells dysfunction which ultimately lead to higher incidence of second primary malignancies (SPMs) and infections [4, 8]. Additionally, BTK inhibitors have been studied in association with risk for secondary malignancies due to its potential interference on immune response [7]. We performed a meta‐analysis of randomised controlled trials (RCTs) to determine the risk of SPMs and nonmelanoma skin cancers (NMSC) in patients with CLL treated with acalabrutinib.

## 2 METHODS

We conducted the systematic review as per the Cochrane Handbook for Systematic Reviews and reported according to the Preferred Reporting Items for Systematic Reviews and Meta‐Analyses (PRISMA) guidelines [9]. A comprehensive literature search was performed through MEDLINE, EMBASE databases and meeting abstracts up to 31st July 2020 using the keywords ‘acalabrutinib OR ACP‐196′ AND ‘CLL’. We reviewed the references of all potential studies for any further relevant studies. We limited the search to ‘humans’ and ‘RCTs’. All studies written in English or non‐English languages were retrieved. The studies that were eligible to be included in the meta‐analysis had to conform with the following characteristics: Phase III RCTs comparing acalabrutinib‐based regimens and a control group in patients with CLL, and RCTs that mention SPM and NMSC as adverse effects. The endpoint of our meta‐analysis was acalabrutinib‐related SPM and NMSC including basal cell carcinoma, squamous cell carcinoma as adverse events.

We summarised the characteristic features of incorporated studies in Table 1 [5, 11]. A total of 833 patients with CLL from two phase III RCTs ([n = 526] in ELEVATE TN, and [n = 307] in ASCEND) were eligible. Studies compared acalabrutinib + obinutuzumab versus acalabrutinib monotherapy versus obinutuzumab + chlorambucil in ELEVATE TN trial and acalabrutinib versus investigator's choice chemotherapy (idelalisib + rituximab or bendamustine + rituximab) in ASCEND trial. Acalabrutinib was administered to 357 patients with treatment‐naïve (TN) CLL in ELEVATE TN study and 154 patients with relapsed or refractory CLL in the ASCEND study. Mantel‐Haenszel method was used to estimate the pooled risk ratio (RR) and risk difference (RD) with 95% confidence interval (CI) for SPM and NMSC. Pooled rates of SPM and NMSC were estimated as follows: we multiplied the median follow‐up duration by the sample size. Crude study‐specific SPM and NMSC rates were then calculated by dividing the number of SPM and NMSC cases by the total number of person‐years follow‐up, respectively. All statistical analyses were performed using the Review Manager, version 5.3 (Nordic Cochrane Centre, Copenhagen, Denmark). Heterogeneity was assessed with *I* ^2^ and Cochran's Q statistic [6]. A ‘*P* value’ of less than .05 was considered significant, and *I* ^2^ > 50% is considered substantially heterogeneous. A RR < 1.0 was in favour of acalabrutinib. Risk of bias for each study was evaluated by Cochrane RevMan 5.3 software. Five main salient biases (selection bias, performance bias, detection bias, attrition bias, reporting bias and others) were categorised and were rated as low, high or unclear risk [6]. Publication bias was assessed by funnel plots.

**TABLE 1:** Characteristics of the studies included in the meta‐analysis

Study (n)	ELEVATE‐ TN trial (n = 526)			ASCEND trial (n = 307)	
Author	Sharman			Ghia	
(Year)	(2020)			(2020)	
Study type	Randomised, multicentre, open‐ label study			Randomised, multicentre, open‐label study	
Study phase	Phase III			Phase III	
Type of cancer	Treatment‐naive chronic lymphocytic leukaemia			Relapsed or refractory chronic lymphocytic leukaemia	
Line of treatment	First line			Second line onwards	
Median follow‐up (months)	28.3			16.1	
Number of patients	178	179	169	154	153
Age					
Median, years (range)	70 (65–75)	70 (66–75)	71 (67–76)	68 (32–89)	67 (34–90)
75 years or older	29.6%	27.9%	29.45%	22%	20%
Number of prior therapies					
Median (range)	Not applicable	Not applicable	Not applicable	1 (1–8)	2 (1–10)
				(Majority 79%‐ 1–2 prior therapies)	(Majority 73%‐ 1–2 prior therapies)
Treatment rendered	Acalabrutinib + Obinutuzumab	Acalabrutinib monotherapy	Obinutuzumab + Chlorambucil	Acalabrutinib monotherapy	Idealalisib + Rituximab (or) Bendamustine+ Rituximab
Median duration of exposure (months)	27.7	27.7	5.6	15.7	5.6‐11.5
Second primary malignancies	35		13	21	7
Nonmelanoma skin cancers	18		4	9	3

## 3 RESULTS

The *I* ^2^ statistic for heterogeneity was low, suggesting homogeneity among RCT, and the fixed effects model was applied. The pooled RR and absolute RD were calculated for SPM and NMSC in both acalabrutinib and control groups. SPM occurred in 56 (10.9%) in the acalabrutinib group compared to 20 (6.2%) in the control group. The RR for SPM was statistically significant at 1.76 (95% CI: 1.09‐2.85; *P* = .02), and the absolute RD was 0.05 (95% CI: 0.01‐0.09; *P* = .02) (Figures 1A and 1B). The pooled rate of SPM among acalabrutinib recipients was 5.32 per 100 person‐years compared to 3.2 per 100 person‐years in participants receiving non‐acalabrutinib therapy.

FIGURE 1A, Pooled risk ratio for second primary malignancies in patients with CLL receiving acalabrutinib versus control. B, Pooled risk difference for second primary malignancies in patients with CLL receiving acalabrutinib versus control. C, Pooled risk ratio for nonmelanoma skin cancers in patients with CLL receiving acalabrutinib versus control. D, Pooled risk difference for nonmelanoma skin cancers in patients with CLL receiving acalabrutinib versus control. E, Pooled risk ratio for melanoma skin cancers in patients with CLL receiving acalabrutinib versus control. F, Pooled risk ratio for lung cancers in patients with CLL receiving acalabrutinib versus control. G, Pooled risk ratio for genitourinary cancers in patients with CLL receiving acalabrutinib versus control. H, Pooled risk ratio for second hematologic malignancies in patients with CLL receiving acalabrutinib versus control

**Figure:**
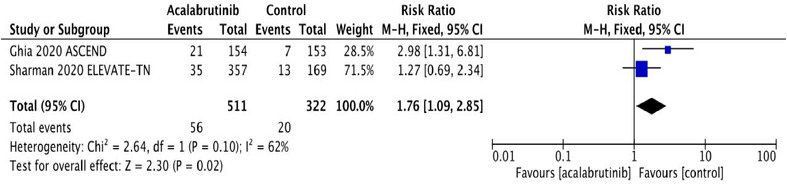

**Figure:**
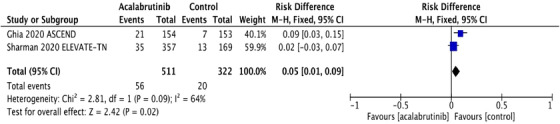

**Figure:**
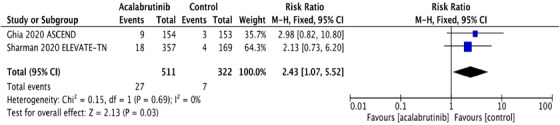

**Figure:**
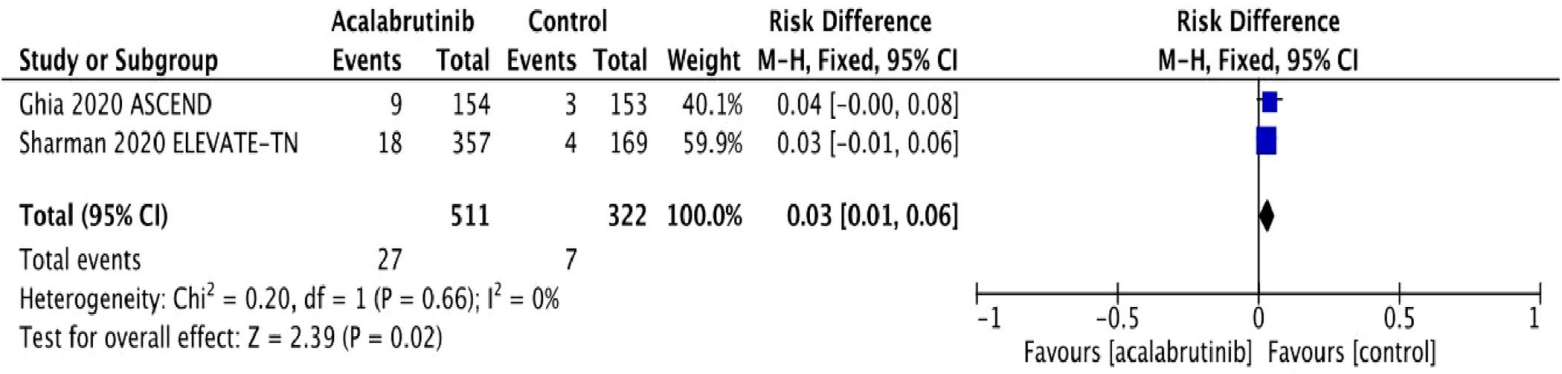

**Figure:**
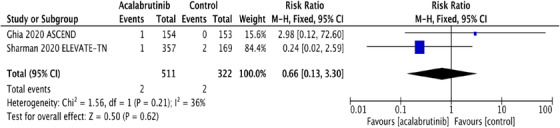

**Figure:**
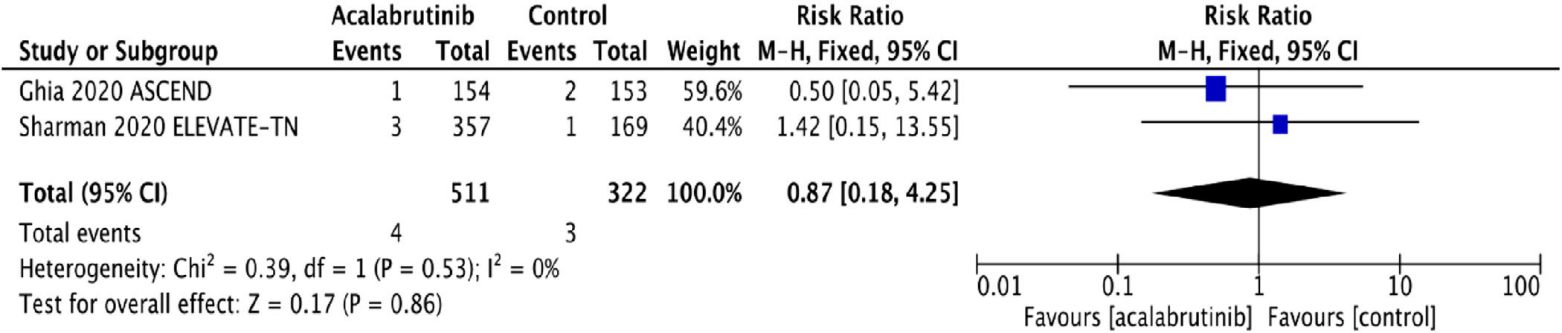

**Figure:**
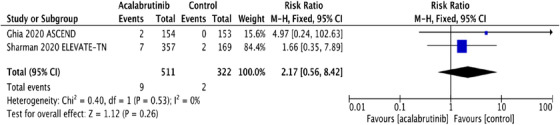

**Figure:**
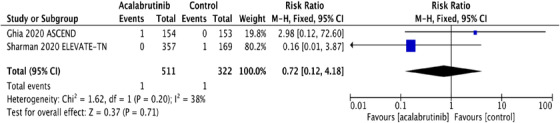

Interestingly, NMSC was the most common SPM observed in both trials, contributing to 48% and 35% of SPM in acalabrutinib and control groups, respectively. NMSC was reported in 27 (5.3%) in the acalabrutinib arm compared to 7 (2.2%) in the control arm. The RR for NMSC was 2.43 (95% CI: 1.07 ‐5.52; *P* = .03**),** and the absolute RD was 0.03 (95% CI: 0.01‐0.06; *P* = .02) (Figures 1C and 1D). The pooled rate of NMSC in patients receiving acalabrutinib containing regimens was 2.56 per 100 person‐years compared to 1.12 per 100 person‐years in the control group. Detailed analysis on the subgroups of SPM excluding NMSC and the differences observed are depicted in Figure 1E‐H.

## 4 DISCUSSION

CLL is the clonal expansion of B cells with the activation in B cell receptor signalling [10]. Second malignancies are associated with worse prognosis in patients with CLL [7]. Acalabrutinib is an oral second generation, small‐molecule irreversible BTK inhibitor, and the remarkable activity of acalabrutinib on progression free survival has been demonstrated in the recent phase III trials (ELEVATE‐TN and ASCEND) [5, 11]. Yet, the risks of acalabrutinib‐related SPM and NMSC remain the areas of concerns.

Our meta‐analysis showed that the incidence of SPM was 4.7 % higher in patients treated with acalabrutinib‐based therapy than control group treated with chemo‐immunotherapy. In other words, acalabrutinib treatment is significantly associated with SPM with RR of 1.76 (95% CI: 1.09‐2.85; *P* = .02). The pooled rate of SPM was 5.32 per 100 person‐years in acalabrutinib group compared to 3.2 per 100 person‐years in the control group. Notably, NMSC was the most common SPM (48% vs 35%) observed in both trials, and patients on acalabrutinib arm were 2.4 times more likely to develop NMSC in patients receiving acalabrutinib containing regimens. The pooled rate of NMSC was 2.56 per 100 person‐years in the acalabrutinib group versus 1.12 per 100 person‐years in the control group.

In the recent single‐centre (The Ohio State University Comprehensive Cancer Centre), retrospective cohort study (from 2009 to 2017), Bond and colleagues reported the incidence of second cancer in ∼700 patients with CLL receiving BTK inhibitors (median age of 61, and 21% receiving acalabrutinib) [1]. After a median follow‐up of 44 months, they observed that 9% were diagnosed with SPM excluding NMSC, and the 3‐year cumulative incidence rate was 16% for NMSC and 7% for other SPM. Mechanistic insight into the other SPM group of the cohort depicted that lung cancer was the most common type of second invasive primary malignancies emerged from BTK inhibitors treatment in the study whereas NMSC was the most common SPM in the ELEVATE‐TN and ASCEND trials. In multivariate analysis, smoking was one of the most significant risk factors whereas higher baseline absolute CD8 count was the protective factor to develop the second invasive primary malignancies in the cohort.

The incidence of acalabrutinib‐related NMSC was not much different in both TN (5%) and relapsed/refractory CLL (5.8%) in the current phase III trials. However, 13.6 % of patients treated with acalabrutinib as salvage treatment reported SPM compared to 9.8% as first‐line treatment which might be potentially contributed by the previous heavy exposure of chemo‐immunotherapy. The differences in patient selection criteria or background patient characteristics may explain the differences in the risks of SPM and NMSC.

It is also notable that median duration of treatment and follow‐up were longer in acalabrutinib‐based regimens than non‐acalabrutinib‐based regimens in both ELEVATE TN and ASCEND trial, which may potentially impact on emergence of treatment‐related adverse events. In ELEVATE TN trial, the median treatment duration was 27.7 months in the acalabrutinib containing regimens, and 5.6 months in the control group. Similarly, in the ASCEND trial, median duration of exposure was 15.7 months for acalabrutinib monotherapy, whereas 5.6‐11.5 months in the control groups.

One of the limitations of our study is that only a limited number of RCTs are currently available. Despite the limited number, we noticed that acalabrutinib containing regimens contributed to higher incidence of SPM (5.32 vs 3.2 per 100 person‐years) with RR of 1.76. Notably, NMSC was the most common SPM, and the incidence was 2.56 vs 1.12 per 100 person‐years. Secondly, median treatment duration and follow‐ups were longer in the acalabrutinib containing regimens in both trials, which may ultimately confound the number of treatment‐related adverse events. Another limitation is that acalabrutinib was administered to patients with relapsed and refractory CLL in ASCEND trial where previous heavy exposure of chemoimmunotherapy may impact on the risk of developing treatment‐related adverse events especially SPM in those groups of patients.

## 5 CONCLUSION

Our meta‐analysis demonstrated that patients on acalabrutinib monotherapy or combination regimens experienced higher incidence of SPM and NMSC compared to non‐acalabrutinib‐based therapy. Further prospective studies are necessary in the future to determine the actual relationship and the potential risk factors. Individual patient level pooled meta‐analysis would provide further detailed and more accurate analyses. Early detection with prompt intervention is warranted.

## CONFLICT OF INTEREST

The authors declare that there is no conflict of interest that could be perceived as prejudicing the impartiality of the research reported.

### AUTHOR CONTRIBUTIONS

Thura W. Htut and Kyaw Z. Thein contributed to the study conception and design. Material preparation, data collection and analysis were performed by Thura W. Htut, Myat M. Han and Kyaw Z. Thein. The first draft of the manuscript was written by Thura W. Htut. Kyaw Z. Thein commented on the manuscript. All authors read and approved the final manuscript.
